# Healthcare utilization and costs following non-fatal powdered and non-powdered firearm injuries for children and youth

**DOI:** 10.1007/s00431-022-04429-4

**Published:** 2022-03-05

**Authors:** Claire de Oliveira, Alison Macpherson, Charlotte Moore Hepburn, Anjie Huang, Rachel Strauss, Ning Liu, Lisa Fiksenbaum, Paul Pageau, David Gomez, Natasha Ruth Saunders

**Affiliations:** 1 ICES, Toronto, Canada; 2 Institute of Health Policy, Management and Evaluation, University of Toronto, Toronto, Canada; 3 Institute for Mental Health Policy Research and Campbell Family Mental Health Research Institute, Centre for Addiction and Mental Health, Toronto, Canada; 4 Centre for Health Economics and Hull York Medical School, University of York, York, UK; 5 School of Kinesiology and Health Science, Faculty of Health, York University, Toronto, Canada; 6 The Hospital for Sick Children, Toronto, ON Canada; 7 Department of Pediatrics, University of Toronto, Toronto, Canada; 8 Temerty Faculty of Medicine, University of Toronto, Toronto, Canada; 9 Child Health Evaluative Sciences, SickKids Research Institute, Toronto, Canada; 10 Department of Emergency Medicine, University of Ottawa, Ottawa, Canada; 11 Department of Surgery, Faculty of Medicine, University of Toronto, Toronto, Canada; 12 Division of General Surgery, St Michael’s Hospital, Unity Health Toronto, Toronto, Canada; 13 Li Ka Shing Knowledge Institute, St Michael’s Hospital, Unity Health Toronto, Toronto, Canada

**Keywords:** Firearms, Injury, Costs, Pediatrics, Youth, Guns, Violence, Suicide, Trauma

## Abstract

Little is known about the healthcare and economic burdens of non-fatal firearm injuries for children/youth beyond the initial admission. This study sought to estimate healthcare utilization and total direct healthcare costs of non-fatal powdered and non-powdered (air gun) firearm injuries 1-year post-injury. Using administrative data from 2003 to 2018 on all children/youth 0–24 years old in Ontario, Canada, a matched 1:2 cohort study was conducted to compare children/youth who experienced powdered and non-powdered firearm injuries with those who did not. Mean and median number of healthcare encounters and costs, and respective 95% confidence intervals (CIs) and interquartile ranges (IQRs), were estimated for both weapon type groups and controls and by intent. Children/youth who experienced a powdered and non-powdered firearm injury had a higher number of healthcare encounters and costs per year than those who did not. Mean 1-year costs for those with powdered and non-powdered firearm injuries were $8825 ($8007–$9643) and $2349 ($2118–$2578), respectively, versus $812 ($567–$1058) and $753 ($594–$911), respectively, for those without. Mean 1-year costs were highest for handgun injuries ($12,875 [95% CI $9941–$15,808]), and for intentional assault-related ($13,498 [$11,843–$15,153]; $3287 [$2213–$4362]), and intentional self-injuries ($14,773 [$6893–$22,652]; $6005 [$2193–$9817]) for both powdered and non-powdered firearm injuries, respectively.

*   Conclusion*: Firearm injuries have substantial healthcare and economic burdens beyond the initial injury-related admission; this should be accounted for when examining the overall impact of firearm injuries.

**Table:** 

**What is Known:** *• Child/youth firearm injuries have significant health and economic burdens.* *• However, existing work has mainly examined healthcare utilization and costs of initial admissions and/or have been limited to single-center studies and no studies have provide cost estimates by weapon type and intent.*	
**What is New:** *• Children/youth who suffered powdered firearm injuries had higher mean healthcare utilization and costs than those with non-powdered firearm injuries as well as comparable healthy children/youth.* *• Mean 1-year costs were highest for handgun injuries ($12,875), and for intentional assault-related ($13,498; $3287), and intentional self-injuries ($14,773; $6005) for powdered and non-powdered firearm injuries, respectively.*

**Supplementary information:**

The online version contains supplementary material available at 10.1007/s00431-022-04429-4.

## Introduction

Firearm injuries remain a major public health concern in high-income countries, such as Canada and the United States (US). On average, one child or youth is injured by a firearm each day in Ontario, while Canada-wide estimates are almost triple that rate [1]. Furthermore, intentional and unintentional injuries, including firearm injuries, are among the top causes of death for young people in Canada [2], while in the US unintentional injuries have been reported as the top cause of death among children and youth [3]. Despite this, most children and youth survive their injury, with many experiencing lasting repercussions post-injury and disability, requiring additional inpatient and outpatient care [4]. Firearm injuries are also associated with large economic burdens. In the US, firearm injuries resulted in $17 billion (2010 USD) in annual healthcare costs across all ages [5]. Average annual costs for the initial admission alone have been reported to be around $622–$735 million (2013 USD) [6, 7]. However, little is known about the healthcare and economic burdens of non-fatal powdered and non-powdered (air gun) firearm injuries for children and youth beyond the initial admission, which are likely to occur at least 1-year post-injury, or at a population-based level, as many studies have been limited to single-center studies [8–10]. Moreover, no work has estimated costs by weapon type and intent. Finally, there has been a paucity of healthcare utilization and cost data from health systems with universal publicly funded health insurance. Cost-of-illness studies provide useful information for decision-makers as they translate the adverse effects of diseases into dollars and help quantify the size of the problem.

Given these gaps, we sought to estimate the healthcare utilization and costs of non-fatal powdered and non-powdered (air gun) firearm injuries 1-year post-injury among a population-based sample of children and youth in Ontario, Canada, overall and by weapon type and intent.

## Materials and methods

### Setting and study design

We conducted a matched cohort study to estimate firearm injury-related healthcare and economic burdens (i.e., utilization and costs) 1-year post-injury among children and youth using administrative data available at ICES, an independent, non-profit research institute located in Toronto, Ontario. The use of these data was authorized under Sect. 45 of Ontario’s Personal Health Information Protection Act, which does not require review by a Research Ethics Board. This observational study was done in accordance with STROBE health data guidelines.

### Data sources

We used administrative healthcare data and population-based databases from Ontario, Canada’s most populous province. Data on institution-based care are captured in the Discharge Abstract Database (medical inpatient hospitalizations, psychiatric inpatient hospitalizations for individuals under the age of 16, and psychiatric inpatient hospitalizations for adults in non-psychiatric designated beds), the Ontario Mental Health Reporting System (all psychiatric inpatient hospitalizations for individuals over the age of 15 in psychiatric designated beds), the Continuing Care Reporting System (continuing and long-term care), and the National Rehabilitation Reporting System (rehabilitation); data on ambulatory care (e.g., emergency department (ED) visits) are recorded in the National Ambulatory Care Reporting System. The Ontario Health Insurance Plan claims database captures data on physician visits and laboratory and diagnostic tests. The Ontario Drug Benefit Program database includes information on outpatient prescription drugs dispensed to individuals covered under the public provincial drug plan (in our analysis, individuals under the age of 65 years living in a long-term care home, a home for special care or a Community Home for Opportunity, receiving professional home and community care services, enrolled in the Trillium Drug Program, or on social assistance). The Home Care Database records visits provided by home care professionals. These databases have been validated and described in the literature [11], and used for costing analyses [12]. See online Supplementary Table A1 for more information.

The Registered Persons Database, a population-based registry maintained by the Ontario Ministry of Health, was used to obtain data on individuals who contacted the healthcare system, such as their date of birth and sex, eligibility for universal healthcare and status changes, and individuals’ postal code of residence, which was used to obtain data on neighborhood-level income quintile and rurality of residence. Data on migrant status were obtained from Immigration, Refugees and Citizenship Canada’s Permanent Resident Database. All datasets were linked using unique encoded identifiers and analyzed at ICES.

### Study population

We included all children and youth 0 to 24 years old with a valid health card number who were discharged alive from the ED or hospital for a non-fatal firearm injury, from April 1, 2003, to March 31, 2018, using International Classification of Diseases, 10th Revision, Clinical Modification (ICD-10-CM) codes for external causes of injury (see online Supplementary Table A2) [13]. Individuals with missing data on census metropolitan area and neighborhood-level income (< 0.3% of the initial sample) were excluded (see Appendix Fig. 1 for inclusion/exclusion flowchart). Children and youth were then divided into two groups—powdered and non-powdered (air gun)—depending on the type of firearm, using validated ICD-10-CA codes (see codes in online Supplementary Table A3) [14]. The hospital admission date was defined as the index date; individuals were followed until the earliest of one year after the index date, their 25th birthday, date of death (where applicable), or March 31, 2019 (end of observation period).

### Analysis

To obtain a *true* estimate of healthcare and economic burdens *due* to a firearm injury, over and above typical healthcare utilization and costs (i.e., the net cost of a firearm injury), as done elsewhere for other conditions [15, 16], children and youth who experienced firearm injuries were hard matched 1:2 (without replacement) to healthy children and youth on sex (male, female), age (+ / − 90 days from birth date), neighborhood income quintile, and geography (census metropolitan area) on the date of firearm-related admission. Healthy children and youth (i.e., those without complex, chronic conditions) were randomly selected (using SAS® function RANUNI) from the Registered Persons Database using the same inclusion/exclusion criteria applied to the study population. Consequently, all children with complex chronic conditions, before and on index date, were excluded from all groups, where complex chronic conditions were defined as “any medical condition that can be reasonably expected to last at least 12 months, unless death intervenes, and involves either several different organ systems or 1 organ system severely enough to require specialty pediatric care and probably some period of hospitalization in a tertiary care centre,” [17] using ICD-9/ICD-10 codes defined elsewhere [18, 19].

We produced the following socio-demographic characteristics for each group (powdered and non-powdered) and respective controls: sex, age at index date, migrant status (non-migrant, immigrant, refugee), neighborhood-level income expressed in quintiles (1, low; 2, medium low; 3, medium; 4, medium high; 5, high), and Rurality Index of Ontario (0–9, large urban; 10–39, small urban; + 40, rural/remote) [20]. Additionally, among those who experienced firearm injuries, we further examined weapon type—hand gun, rifle, and undetermined/unspecified for powdered firearms—and intent of injury for both powdered and non-powdered firearms—unintentional, intentional assault, intentional self-injury, legal intervention, and undetermined—using validated ICD-10-CA codes (see codes in online Supplementary Table A3) [14]. Differences between groups were examined using standardized mean differences (SMDs), where SMDs > 0.10 are considered large [21].

### Healthcare utilization

We estimated the mean and median number (and respective 95% confidence intervals (Cis), standard deviation (SD), and interquartile range (IQR)) of medical inpatient hospitalizations, psychiatric inpatient hospitalizations, ED visits, outpatient physician visits, and home care visits in the 1-year post-injury (including firearm injury admission) for powdered and non-powdered groups and their respective healthy controls. We also estimated the mean and median length of stay (and respective 95% CIs, SDs, and IQRs) for inpatient medical and psychiatric hospitalizations, which occurred in the 1-year post-injury.

### Healthcare costs

Healthcare costs were estimated from the third-party public payer perspective (i.e., the Ontario Ministries of Health and Long-term Care) using a cost algorithm available at ICES [12], which employs a bottom-up/micro-costing approach to cost services at the individual patient level. This approach identifies individual episodes of care or utilization in the healthcare system and attaches unit costs (or, where lacking, amounts/prices paid) to each one. Given Ontario’s public healthcare insurance system, providers in a private marketplace rarely set prices; therefore, amounts paid by the Ministries of Health and Long-term Care were used. In cases where individual unit costs were unavailable (e.g., for institutional care settings, such as long-term care homes), a top-down approach, which allocates corporate aggregate costs to individual visits or episodes of care, was employed. Further details on the costing methodology can be found elsewhere [12]. The costs captured by the algorithm account for > 90% of all government paid health services [22]. Costs were aggregated into medical inpatient hospitalizations, psychiatric inpatient hospitalizations, ED visits, outpatient physician visits, home care visits, and other care (i.e., other institution-based care, other ambulatory care, and outpatient prescription drugs). We estimated total direct mean and median healthcare costs 1-year post-injury (including firearm injury admission) for each weapon type group and controls, overall and by health service, and the mean between-group difference, and respective 95% CIs, SDs and IQRs. We also estimated mean and between-group costs, overall and by health service, and respective 95% CIs, by intent. All costs were expressed in 2017 Canadian dollars (1 CAD = 0.69 EUR on February 18, 2022) [23].

We estimated the between-group utilization and cost differences using generalized estimating equations, where the outcome was utilization or cost and the predictor was firearm injury (yes/no), to account for the clustering effect within matched individual sets [24].

## Results

Nearly all (4188/4189) children and youth who experienced a firearm injury had 2 controls. Children and youth who experienced a firearm injury were mainly comprised of males (90%); however, those with powdered firearm injuries were slightly older and more concentrated in low-income neighborhoods than those with non-powdered firearm injuries (mean age 19 versus 17, and 41% versus 26%, respectively) (Table 1). Although most individuals lived in large urban areas, those with powdered firearms were over-represented in more urban areas (78% versus 48%). Children and youth who experienced firearm injuries and those who did not were well matched on all socio-demographic and geographic characteristics (SMD < 0.10) except migrant status; moreover, those with powdered firearm injuries were more likely to be immigrants or refugees compared to those with non-powdered firearm injuries (12% and 7% versus 4% and 1%, respectively). Among those who experienced powdered firearm injuries, most were due to undetermined/unspecified firearms (75%); the most common intent was unintentional (58%), followed by intentional assault (34%). For those who experienced non-powdered firearm injuries, the most common intent was unintentional (79%).

**Table 1:** Baseline socio-demographic characteristics of children and youth who experienced a powdered and non-powdered firearm injury (cases) and respective group-matched healthy children and youth (controls)

Variable	Powdered firearms*			Non-powdered (air gun) firearms		
	Cases	Controls	SMD	Cases	Controls	SMD
Overall	*N* = 2001	*N* = 4002		*N* = 2188	*N* = 4375	
Sex						
Female	207 (10.3%)	414 (10.3%)	0.000	228 (10.4%)	456 (10.4%)	0.000
Male	1794 (89.7%)	3588 (89.7%)	0.000	1960 (89.6%)	3919 (89.6%)	0.000
Age, years						
0–12	124 (6.2%)	255 (6.4%)	0.007	378 (17.3%)	760 (17.4%)	0.002
13–17	450 (22.5%)	902 (22.5%)	0.001	841 (38.4%)	1683 (38.5%)	0.000
18–24	1427 (71.3%)	2845 (71.1%)	0.005	969 (44.3%)	1932 (44.2%)	0.002
Mean ± SD	18.95 ± 3.87	18.94 ± 3.88	0.003	16.53 ± 4.30	16.52 ± 4.30	0.001
Median (IQR)	19 (17–22)	19 (17–22)	0.003	17 (14–20)	17 (14–20)	0.001
Neighborhood income quintile						
1, low	813 (40.6%)	1626 (40.6%)	0.000	568 (26.0%)	1136 (26.0%)	0.000
2, medium low	433 (21.6%)	866 (21.6%)	0.000	462 (21.1%)	924 (21.1%)	0.000
3, medium	354 (17.7%)	708 (17.7%)	0.000	409 (18.7%)	817 (18.7%)	0.000
4, medium high	243 (12.1%)	486 (12.1%)	0.000	440 (20.1%)	880 (20.1%)	0.000
5, high	158 (7.9%)	316 (7.9%)	0.000	309 (14.1%)	618 (14.1%)	0.000
Migrant status						
Non-migrant	1630 (81.5%)	3164 (79.1%)	0.060	2075 (94.8%)	4006 (91.6%)	0.130
Immigrant	239 (11.9%)	675 (16.9%)	0.141	82 (3.7%)	271 (6.2%)	0.113
Refugee	132 (6.6%)	163 (4.1%)	0.112	31 (1.4%)	98 (2.2%)	0.061
Rurality Index of Ontario						
0–9, large urban	1564 (78.2%)	3101 (77.5%)	0.016	1043 (47.7%)	2233 (51.0%)	0.067
10–39, small urban	277 (13.8%)	604 (15.1%)	0.036	752 (34.4%)	1329 (30.4%)	0.086
40 + , rural/remote	122 (6.1%)	249 (6.2%)	0.005	312 (14.3%)	678 (15.5%)	0.035
Missing	38 (1.9%)	48 (1.2%)	0.057	81 (3.7%)	135 (3.1%)	0.034
Weapon type						
Handguns	263 (13.1%)	–	–	–	–	–
Rifles	216 (10.8%)	–	–	–	–	–
Undetermined or unspecified	1496 (74.8%)	–	–	–	–	–
Non-powdered (air gun) firearms	–	–	–	2188 (100.0%)	–	–
Intent						
Unintentional	1166 (58.3%)	–	–	1732 (79.2%)	–	–
Intentional assault	689 (34.4%)	–	–	219 (10.0%)	–	–
Intentional self-injury	20 (1.0%)	–	–	28 (1.3%)	–	–
Legal intervention	26 (1.3%)	–	–	–	–	–
Undetermined	100 (5.0%)	–	–	209 (9.6%)	–	–

*SMD* standardized mean difference, *SD* standard deviation, *IQR* interquartile range

^*^Powdered firearms include handguns, rifles, and undetermined or unspecified firearms; between-group SMDs for each age group were close to 0 and thus rounded down

**Table 2:** Healthcare utilization in the 365 days post-injury (including index injury date) of children and youth who experienced a powdered and non-powdered firearm injury (cases), respective group-matched healthy children and youth (controls) and mean between-group difference

Powdered firearms*				
Health service	Cases	Controls	SMD	Between-group difference
	*N* = 2001	*N* = 4002		
Inpatient medical hospitalizations				
Mean, 95% CI ± SD	0.5 (0.4–0.5) ± 0.6	0.0 (0.0–0.0) ± 0.1	0.97	0.4 (0.4–0.5)
Median (IQR)	0 (0–1)	0 (0–0)		
Length of stay				
Mean, 95% CI ± SD	6.4 (5.7–7.1) ± 9.6	6.7 (3.9–9.5) ± 8.4	0.03	− 0.3 (− 3.2–2.6)
Median (IQR)	4 (2–7)	3 (2–6)		
Inpatient psychiatric hospitalizations				
Mean, 95% CI ± SD	0.0 (0.0–0.0) ± 0.2	0.0 (0.0–0.0) ± 0.1	0.12	0.0 (0.0–0.0)
Median (IQR)	0 (0–0)	0 (0–0)		
Length of stay				
Mean, 95% CI ± SD	40.0 (− 0.8–80.7) ± 141.1	47.7 (− 4.8–100.1) ± 151.1	0.05	− 7.7 (− 74.2–58.7)
Median (IQR)	9 (4–27)	7 (3–14)		
Emergency department visits				
Mean, 95% CI ± SD	1.5 (1.4–1.6) ± 1.7	0.3 (0.3–0.4) ± 0.9	0.88	1.2 (1.1–1.3)
Median (IQR)	1 (1–2)	0 (0–0)		
Outpatient physician visits				
Mean, 95% CI ± SD	5.6 (5.3–5.9) ± 6.2	2.3 (2.2–2.4) ± 3.8	0.64	3.3 (3.0–3.6)
Median (IQR)	4 (2–7)	1 (0–3)		
Home care visits				
Mean, 95% CI ± SD	1.9 (1.5–2.3) ± 9.9	0.1 (0.1–0.1) ± 1.6	0.26	1.8 (1.4–2.3)
Median (IQR)	0 (0–0)	0 (0–0)		
Non-powdered (air gun) firearms				
Health service	Cases	Controls	SMD	Between-group difference
	*N* = 2188	*N* = 4375		
Inpatient medical hospitalizations				
Mean, 95% CI ± SD	0.1 (0.1–0.1) ± 0.3	0.0 (0.0–0.0) ± 0.2	0.24	0.1 (0.0–0.1)
Median (IQR)	0 (0–0)	0 (0–0)		
Length of stay				
Mean, 95% CI ± SD	3.2 (2.8–3.6) ± 2.6	4.5 (3.2–5.7) ± 5.3	0.31	− 1.2 (− 2.6–0.1)
Median (IQR)	2 (2–4)	3 (2–5)		
Inpatient psychiatric hospitalizations				
Mean, 95% CI ± SD	0.0 (0.0–0.0) ± 0.2	0.0 (0.0–0.0) ± 0.1	0.09	0.0 (0.0–0.0)
Median (IQR)	0 (0–0)	0 (0–0)		
Length of stay				
Mean, 95% CI ± SD	21.9 (12.5–31.4) ± 31.3	33.9 (3.3–64.5) ± 88.3	0.18	− 11.9 (− 43.9–20.0)
Median (IQR)	9 (4–24)	5 (4–25)		
Emergency department visits				
Mean, 95% CI ± SD	2.0 (1.9–2.0) ± 1.8	0.4 (0.4–0.5) ± 1.0	1.04	1.5 (1.4–1.6)
Median (IQR)	1 (1–2)	0 (0–1)		
Outpatient physician visits				
Mean, 95% CI ± SD	3.9 (3.7–4.1) ± 4.8	1.9 (1.8–2.0) ± 3.1	0.48	2 (1.7–2.2)
Median (IQR)	3 (1–5)	1 (0–3)		
Home care visits				
Mean, 95% CI ± SD	0.3 (0.1–0.4) ± 4.3	0.1 (0.1–0.2) ± 2.5	0.04	0.1 (− 0.1–0.3)
Median (IQR)	0 (0–0)	0 (0–0)		

*SMD* standardized mean difference, *CI* confidence interval, *SD* standard deviation, *IQR* interquartile range

^*^Powdered firearms include handguns, rifles, and undetermined or unspecified firearms

Children and youth who experienced powdered and non-powdered firearm injuries had a higher mean number of healthcare encounters in the year post-injury than those who did not, especially for outpatient physician services—5.6 (5.3–5.9) and 3.9 (3.7–4.0) versus 2.3 (2.2–2.4) and 1.9 (1.8–2.0), respectively (Table 2).

Total direct mean and median 1-year post-injury healthcare costs for children and youth who experienced powdered and non-powdered firearm injuries were $8825 ($8007–$9643) and $2861 ($886–$9347), and $2349 ($2118–$2580) and $984 ($490–$2249), respectively; the corresponding estimates for comparable healthy children and youth were $812 ($567–$1058) and $123 ($16–$380), and $2349 ($2118–$2580) and $984 ($490–$2249), respectively (Table 3). The between-group mean cost difference was $8013 ($7157–$8868) and $1596 ($1322–$1871), respectively. Overall, costs were mostly due to inpatient medical hospitalizations (41%) and outpatient physician visits (26%) for powdered firearm injuries and outpatient physician visits (32%) and emergency department visits (20%) for non-powdered firearm injuries; costs were higher for powdered firearms injuries for all health services except same-day surgeries ($111 versus $159).

**Table 3:** Total healthcare costs (in 2017 CAD) in the 365 days post-injury (including index injury date) of children and youth who experienced a powdered and non-powdered firearm injury (cases), respective group-matched healthy children and youth (controls) and mean between-group difference

Powdered firearms*				
Health service	Cases	Controls	SMD	Between-group difference
	*N* = 2001	*N* = 4002		
Inpatient medical hospitalizations				
Mean, 95% CI ± SD	3619 (3157–4082) ± 10,554	59 (32–86) ± 871	0.48	3560 (3097–4024)
Median (IQR)	0 (0–4049)	0 (0–0)		
Inpatient psychiatric hospitalizations				
Mean ± SD	424 (184–665) ± 5485	288 (58–517) ± 7418	0.02	137 (− 196–4670)
Median (IQR)	0 (0–0)	0 (0–0)		
Emergency department visits				
Mean ± SD	694 (661–727) ± 758	76 (69–84) ± 242	1.1	618 (584–652)
Median (IQR)	423 (168–966)	0 (0–0)		
Same-day surgery				
Mean ± SD	111 (92–131) ± 445	21 (15–27) ± 192	0.26	90 (70–111)
Median (IQR)	0 (0–0)	0 (0–0)		
Outpatient physician visits				
Mean ± SD	2263 (2108–2419) ± 3545	240 (220–259) ± 645	0.79	2024 (1868–2180)
Median (IQR)	954 (355–2669)	75 (0–227)		
Home care visits				
Mean ± SD	321 (267–374) ± 1225	15 (9–22) ± 210	0.35	305 (251–360)
Median (IQR)	0 (0–0)	0 (0–0)		
Other care				
Mean ± SD	1392 (1119–1664) ± 6214	114 (95–132) ± 603	0.29	1278 (1005–1551)
Median (IQR)	94 (0–865)	8 (0–75)		
Total cost				
Mean ± SD	8825 (8007–9643) ± 18,684	812 (567–1058) ± 7941	0.56	8013 (7157–8868)
Median (IQR)	2861 (886–9347)	123 (16–380)		
Non-powdered (air gun) firearms				
Health service	Cases	Controls	SMD	Between-group difference
	*N* = 2188	*N* = 4375		
Inpatient medical hospitalizations				
Mean, 95% CI ± SD	280 (213–347) ± 1590	125 (58–192) ± 2265	0.08	155 (61–250)
Median (IQR)	0 (0–0)	0 (0–0)		
Inpatient psychiatric hospitalizations				
Mean ± SD	281 (145–417) ± 3240	127 (10–245) ± 3971	0.04	15 (− 26–333)
Median (IQR)	0 (0–0)	0 (0–0)		
Emergency department visits				
Mean ± SD	467 (444–490) ± 548	94 (85–102) ± 275	0.86	374 (350–398)
Median (IQR)	290 (161–562)	0 (0–108)		
Same-day surgery				
Mean ± SD	159 (135–183) ± 566	33 (25–40) ± 252	0.29	126 (101–151)
Median (IQR)	0 (0–0)	0 (0–0)		
Outpatient physician visits				
Mean ± SD	744 (691–798) ± 1270	229 (209–248) ± 668	0.51	516 (460–572)
Median (IQR)	401 (201–781)	69 (0–218)		
Home care visits				
Mean ± SD	40 (19–62) ± 510	19 (12–25) ± 233	0.05	22 (− 1–44)
Median (IQR)	0 (0–0)	0 (0–0)		
Other care				
Mean ± SD	378 (341–414) ± 876	127 (106–149) ± 724	0.31	250 (209–292)
Median (IQR)	82 (0–416)	15 (0–91)		
Total cost				
Mean ± SD	2349 (2118–2580) ± 5507	753 (594–911) ± 5360	0.29	1596 (1322–1871)
Median (IQR)	984 (490–2249)	140 (41–424)		

*SMD* standardized mean difference, *CI* confidence interval, *SD* standard deviation, *IQR* interquartile range

^*^Powdered firearms include handguns, rifles, and undetermined or unspecified firearms

Direct healthcare costs differed by weapon type (Table 4); these were highest for handguns ($12,875 [95% $9941–$15,808]), and lowest for non-powdered firearms ($2349 [95% CI $2118–$2580]). Medical inpatient hospitalizations (40–42%), outpatient physician visits (24–27%) and other costs (10–24%) made up most of the cost for powdered firearm injuries; outpatient physician visits (32%), ED visits (20%), and other costs (16%) made up most of the cost for non-powdered firearm injuries.

**Table 4:** Total healthcare costs (in 2017 CAD) in the 365 days post-injury (including index injury date) of children and youth who experienced a firearm injury (cases), matched healthy children and youth (controls) and mean between-group difference, and respective 95% confidence intervals, by weapon type

	Cases, mean estimate, 95% CI	Controls, mean estimate, 95% CI	Between-group difference, mean estimate, 95% CI
Powdered firearms			
Handguns			
Inpatient medical hospitalizations	5193 (3965–6421)	88 (0–189)	5105 (3871–6338)
Inpatient psychiatric hospitalizations	175 (0–415)	553 (0–1316)	− 379 (− 1179–422)
Emergency department visits	849 (749–949)	77 (57–96)	772 (668–877)
Same-day surgery	84 (39–130)	16 (0–32)	68 (20–117)
Outpatient physician visits	3122 (2647–3597)	258 (205–312)	2863 (2385–3342)
Home care visits	335 (194–476)	15 (0–30)	320 (178–462)
Other care	3117 (1662–4572)	158 (60–257)	2959 (1500–4418)
Total cost	12,875 (9941–15,808)	1165 (372–1959)	11,709 (8661–14,758)
Rifles			
Inpatient medical hospitalizations	3696 (1355–6038)	34 (0–73)	3662 (1321–6004)
Inpatient psychiatric hospitalizations	775 (188–1361)	627 (0–1798)	148 (− 1169–1464)
Emergency department visits	686 (578–794)	86 (64–108)	600 (494–707)
Same-day surgery	158 (91–224)	21 (5–38)	136 (67–205)
Outpatient physician visits	2150 (1638–2661)	202 (160–244)	1948 (14,367–2459)
Home care visits	403 (244–561)	9 (0–21)	394 (236–552)
Other care	855 (566–1143)	9 (58–135)	758 (466–1051)
Total cost	8722 (5673–11,771)	1075 (0–2266)	7647 (4363–10,931)
Undetermined or unspecified			
Inpatient medical hospitalizations	3351 (2883–3819)	59 (28–89)	3292 (2822–3762)
Inpatient psychiatric hospitalizations	277 (103–452)	195 (0–413)	83 (197–363)
Emergency department visits	670 (633–708)	75 (66–84)	596 (558–634)
Same-day surgery	109 (87–132)	22 (15–29)	87 (64–111)
Outpatient physician visits	2136 (1963–2310)	242 (218–266)	1894 (1719–2069)
Home care visits	310 (247–373)	16 (8–24)	294 (230–358)
Other care	1162 (910–1414)	109 (92–126)	1053 (800–1305)
Total cost	8016 (7199–8833)	718 (476–960)	7298 (6445–8151)
Non-powdered (air gun) firearms			
Inpatient medical hospitalizations	280 (213–347)	125 (58–192)	155 (61–250)
Inpatient psychiatric hospitalizations	281 (145–416)	127 (10–245)	153 (− 26–333)
Emergency department visits	467 (444–490)	94 (85–102)	374 (50–398)
Same-day surgery	159 (135–183)	33 (25–40)	126 (101–151)
Outpatient physician visits	744 (691–798)	229 (209–248)	516 (460–572)
Home care visits	40 (19–62)	19 (12–25)	22 (− 1–44)
Other care	378 (341–414)	127 (106–149)	250 (209–292)
Total cost	2349 (2118–2580)	753 (594–911)	1596 (1322–1871)

*CI* confidence interval

Direct healthcare costs also differed by intent (Table 5). Among powdered firearm injuries, costs were highest for legal intervention firearms ($15,262 [95% CI $1135–$29,389]) and intentional self-injury ($14,773 [$6893–$22,652]) (though these groups only included 26 and 20 individuals, respectively), followed by intentional assault $13,498 ($11,843–$15,153). Among non-powdered firearm injuries, costs were highest for intentional self-harm injury ($6005 [$2193–$9817]), followed by intentional assault ($3287 [$2213–$4362]). Medical inpatient hospitalizations (39–42%) and outpatient physician visits (26%) made up most costs for powdered firearm injuries of all intents, except those for legal intervention and intentional self-inflicted firearm injuries, where most costs were due to psychiatric inpatient hospitalizations (56% and 58%, respectively). For non-powdered firearm injuries, outpatient physician visits (28–33%), psychiatric inpatient hospitalizations (20–44%), and emergency department visits (10–21%) made up most costs for all intents.

**Table 5:** Total healthcare costs (in 2017 CAD) in the 365 days post-injury (including index injury date) of children and youth who experienced a firearm injury (cases), matched healthy children and youth (controls) and mean between-group difference, and respective 95% confidence intervals, by intent

Powdered firearms*			
	Cases, mean estimate, 95% CI	Controls, mean estimate, 95% CI	Between-group difference, mean estimate, 95% CI
Unintentional			
	*N* = 1166	*N* = 2332	
Inpatient medical hospitalizations	2582 (1948–3216)	68 (26–109)	2514 (1879–3150)
Inpatient psychiatric hospitalizations	248 (103–394)	480 (86–873)	− 231 (− 652–189)
Emergency department visits	584 (548–620)	82 (71–92)	502 (465–539)
Same-day surgery	109 (84–134)	19 (12–25)	90 (65–116)
Outpatient physician visits	1606 (1443–1770)	243 (215–272)	1363 (1197–1529)
Home care visits	228 (182–274)	20 (10–30)	208 (161–255)
Other care	785 (631–940)	110 (91–129)	675 (519–831)
Total cost	6143 (5263–7022)	1022 (605–1439)	5121 (4147–6095)
Intentional assault			
	*N* = 689	*N* = 1378	
Inpatient medical hospitalizations	5764 (5007–6522)	51 (18–84)	5713 (4955–6472)
Inpatient psychiatric hospitalizations	230 (0–541)	18 (0–40)	212 (− 100–523)
Emergency department visits	904 (835–972)	67 (55–78)	837 (768–907)
Same-day surgery	113 (78–147)	25 (13–37)	88 (52–124)
Outpatient physician visits	3494 (3170–3818)	234 (206–262)	3261 (2937–3584)
Home care visits	504 (372–636)	8 (0–16)	496 (363–628)
Other care	2489 (1764–3214)	115 (75–156)	2374 (1647–3100)
Total cost	13,498 (11,843–15,153)	518 (431–606)	12,980 (11,325–14,634)
Intentional self-injury			
	*N* = 20	*N* = 40	
Inpatient medical hospitalizations	0 (0–0)	73 (0–212)	− 73 (− 212–66)
Inpatient psychiatric hospitalizations	8534 (3931–13,137)	0 (0–0)	8534 (3931–13,137)
Emergency department visits	916 (523–1309)	146 (30–262)	770 (391–1149)
Same-day surgery	271 (0–567)	56 (0–135)	215 (− 100–530)
Outpatient physician visits	3566 (1743–5389)	372 (71–674)	3194 (1292–5095)
Home care visits	105 (0–305)	16 (0–47)	89 (− 115–293)
Other care	1381 (0–2922)	349 (0–721)	1032 (− 566–2630)
Total cost	14,773 (6893–22,652)	1012 (100–1923)	13,761 (5714–21,808)
Legal intervention			
	*N* = 26	*N* = 52	
Inpatient medical hospitalizations	2523 (695–4352)	0 (0–0)	2523 (695–4352)
Inpatient psychiatric hospitalizations	8509 (0–22,698)	136 (0–398)	8373 (− 5831–22,577)
Emergency department visits	561 (378–743)	70 (9–131)	491 (305–677)
Same-day surgery	118 (0–281)	6 (0–16)	112 (− 53–277)
Outpatient physician visits	1848 (948–2749)	204 (89–319)	1645 (723–2566)
Home care visits	98 (0–245)	30 (0–87)	68 (− 91–229)
Other care	1605 (496–2714)	55 (12–97)	1550 (436–2665)
Total cost	15,262 (1135–29,389)	500 (0–1002)	14,762 (612–28,912)
Undetermined			
	*N* = 100	*N* = 200	
Inpatient medical hospitalizations	1945 (675–3215)	27 (0–80)	1918 (645–3190)
Inpatient psychiatric hospitalizations	92 (− 199)	0 (0–0)	92 (− 15–199)
Emergency department visits	524 (408–641)	61 (36–86)	463 (351–576)
Same-day surgery	99 (16–182)	20 (0–49)	79 (− 10–167)
Outpatient physician visits	1294 (851–1738)	220 (153–288)	1074 (645–1503)
Home care visits	240 (90–389)	0 (0–0)	240 (90–389)
Other care	849 (0–1736)	114 (49–179)	735 (− 156–1625)
Total cost	5043 (2441–7644)	443 (278–608)	4600 (2017–7183)
Non-powdered (air gun) firearms			
	Cases, mean estimate, 95% CI	Controls, mean estimate, 95% CI	Between-group difference, mean estimate, 95% CI
Unintentional			
	*N* = 1732	*N* = 3464	
Inpatient medical hospitalizations	246 (194–299)	124 (42–206)	122 (25–220)
Inpatient psychiatric hospitalizations	143 (33–253)	142 (0–287)	1 (− 181–183)
Emergency department visits	448 (424–471)	96 (86–106)	352 (326–377)
Same-day surgery	164 (138–190)	35 (26–44)	129 (102–157)
Outpatient physician visits	694 (640–747)	228 (205–250)	466 (410–522)
Home care visits	45 (18–72)	21 (13–29)	24 (− 4–52)
Other care	378 (337–419)	132 (106–159)	246 (198–293)
Total cost	2118 (1905–2331)	778 (585–971)	1340 (1062–1617)
Intentional assault			
	*N* = 219	*N* = 438	
Inpatient medical hospitalizations	381 (186–576)	137 (6–268)	244 (6–481)
Inpatient psychiatric hospitalizations	791 (0–1,62)	0 (0–0)	791 (− 41–1622)
Emergency department visits	545 (467–623)	96 (70–121)	450 (370–529)
Same-day surgery	140 (64–215)	26 (6–46)	113 (36–191)
Outpatient physician visits	975 (769–1181)	256 (182–331)	719 (502–935)
Home care visits	21 (0–42)	16 (0–35)	5 (− 24–34)
Other care	435 (299–571)	125 (84–166)	310 (168–452)
Total cost	3287 (2213–4362)	656 (408–905)	2631 (1524–3738)
Intentional self-injury			
	*N* = 28	*N* = 55	
Inpatient medical hospitalizations	507 (10–1005)	59 (0–173)	448 (− 70–966)
Inpatient psychiatric hospitalizations	2632 (292–4972)	0 (0–0)	2632 (292–4972)
Emergency department visits	596 (384–808)	93 (37–150)	503 (288–718)
Same-day surgery	258 (0–545)	0 (0–0)	258 (− 29–544)
Outpatient physician visits	1750 (710–2790)	178 (87–268)	1572 (517–2627)
Home care visits	0 (0–0)	0 (0–0)	0 (0–0)
Other care	262 (84–441)	56 (29–84)	206 (32–380)
Total cost	6005 (2193–9817)	387 (164–609)	5619 (1769–9469)
Undetermined			
	*N* = 209	*N* = 418	
Inpatient medical hospitalizations	423 (0–922)	125 (19–231)	298 (− 213–810)
Inpatient psychiatric hospitalizations	571 (18–1125)	154 (0–418)	417 (− 199–1033)
Emergency department visits	533 (427–639)	71 (50–93)	462 (356–567)
Same-day surgery	122 (42–202)	26 (9–44)	96 (13–179)
Outpatient physician visits	788 (575–1001)	22 (160–263)	576 (356–797)
Home care visits	27 (2–51)	2 (0–5)	25 (− 1–50)
Other care	331 (224–437)	101 (74–129)	230 (124–336)
Total cost	2795 (1732–3857)	692 (50–1034)	2103 (980–3225)

*CI* confidence interval

^*^Powdered firearms include handguns, rifles, and undetermined or unspecified firearm

## Discussion

Using a population-based sample of children and youth, we found that healthcare utilization was higher for all health services among those who experienced non-fatal powdered and non-powdered firearm injuries in the 1-year post-injury compared to healthy children and youth. Total mean and median costs for powdered and non-powdered firearm injuries were $8,8245, and $2349, while for healthy controls, these were $812 and $753, respectively, thus providing mean powdered and non-powdered firearm-related cost estimates of $1278 and $1596, respectively. One-year healthcare costs were highest for powdered firearm injuries, namely handguns, and intentional assault and self-injuries for both powdered and non-powdered firearm injuries. Non-fatal firearm injuries contribute to high utilization and costs beyond the initial injury; this should be accounted for when examining the broader impact of these injuries as many survivors require lasting medical care.

Our findings align with other research. One study, which compared youth who experienced firearm injuries and those who experienced motor vehicle accidents, found that the former were more likely to be admitted to an intensive care unit (adjusted odds ratio (aOR) 6.7, 95% CI 5.9–7.7) and have longer lengths of stays (aOR 2.2, 95% CI 1.9–2.6) than the latter [25]. Moreover, children with firearm injuries had more return visits, and subsequent inpatient admission within 3 days (aOR 3.4, 95% CI 2.1–5.5) and 1 year (aOR 2.5, 95% CI 2.1–2.9) post-injury. Similar results were found elsewhere, where patients surviving firearm injuries had a substantially higher risk of subsequent hospitalizations than pedestrian or occupant motor vehicle accident injuries [26].

Regarding the economic burden, most literature has generally estimated costs of initial admissions or readmissions. Using 2010–2014 data from the Nationwide Readmissions Database, one study estimated a median cost of $12,619 for the initial injury-related hospitalization, while the median cost of the first readmission within 30 days and 1 year post-discharge was $7804 and $8451, respectively [8]. Another study using the Nationwide Emergency Department Sample 2006–2014 found that median ED visit and inpatient charges (2018 USD) were $2445 (IQR: $1318–$5191) and $44,966 (IQR: $21,156–$91,771), respectively [9]. Other work, examining the burden of musculoskeletal firearm injuries in children with and without concomitant intra-cavitary injuries, found a median cost of initial hospitalization and additional encounters and outpatient follow-up of $16,356 (IQR: $8246–$30,972) per patient, though data were from a single center [10]. Our cost estimates are substantially lower than those reported elsewhere, likely because most studies are from the US, where gun violence is more prevalent and healthcare is more costly. Few studies have studied firearm-related injury costs over longer periods of time (e.g., 1 year) [4], estimated other costs beyond those related to the initial admission (e.g., outpatient physician and home care visits costs), or examined non-powdered firearm injuries, which have substantial health and healthcare system impacts (albeit to a lesser extent than powdered firearm injuries).

We found that 1-year costs were highest for firearm injuries due to handguns, followed by rifles, and lowest for non-powdered firearms. The costs of handgun and rifle injuries were mainly due to medical inpatient hospitalizations and outpatient physician visits; for non-powdered firearm injuries, these were mainly due to outpatient physician and ED visits. The cost difference is likely due to treatment differences—handgun- and rifle-related injuries typically require specialized care (e.g., surgery) and longer hospitalizations [10]. Nonetheless, despite a lower mean cost, the total economic burden of non-powdered firearms was substantially higher than that for handguns and rifles ($5,189,148 versus $3,430,467 and $1,908,906, respectively). Moreover, these costs are largely preventable. Costs were also greater for assault-related injuries and those due to suicidal intent, where costs were mostly due to medical and psychiatric inpatient hospitalizations, respectively.

Firearm-related injuries are among the top causes of death and disability for children. Furthermore, child and youth firearm injuries can have significant healthcare and economic burdens with potential long-term effects such as lifelong physical impairments and reduced quality of life. Unintentional injuries, and related costs, could likely be prevented with the implementation of safety training, safe firearm storage practices, and appropriate supervision. Additionally, strong and effective legalization around firearms control and awareness campaigns around their use, and policies that address mental health and self-harm among children and youth should be considered. This is particularly relevant for non-powdered firearms; given the substantial healthcare and economic burden of these firearms (compared to healthy children), consideration should be given to regulating their use.

### Strengths and limitations

We undertook a population-based study examining all children and youth in Ontario and were able to estimate the economic burden due to powdered and non-powdered firearm injuries in the year post-injury, which included most direct costs covered under the public healthcare system and, for the first time, provided cost estimates by weapon type and intent. Nonetheless, there were many cases of unknown weapon type. We were not able to account for costs of specialized community-based drug and alcohol services. Moreover, although a small proportion (6%), not all children/youth had a full year of cost data, which may have biased our cost estimates. We were not able to examine the data by race/ethnicity; however, prior research has shown that immigrants in Ontario, particularly those from Africa and Central American countries, are disproportionately assaulted by firearms compared to non-immigrants [1]. We did not estimate firearm-related costs incurred in other sectors (e.g., education sector). We also did not examine other direct costs (e.g., out-of-pocket costs) or indirect costs, i.e., productivity losses due to caregiver absenteeism associated with a child’s hospitalization or productivity losses for older youth employed at time of injury. Finally, we only estimated 1-year post-injury costs; future work should seek to estimate costs for longer periods to understand the long-term impact of firearm injuries.

## Conclusion

Firearm injuries have substantial healthcare and economic burdens beyond initial injury-related admissions. We found that children and youth had higher healthcare utilization and costs than comparable, healthy children and youth in the 1-year post-injury. One-year mean costs were highest for powdered firearm injuries, specifically handguns, and for intentional assault and self-injuries for both powdered and non-powdered firearm injuries. The implementation of legalization around firearms control and awareness campaigns around their use, and policies that address child and youth mental health and self-harm behavior may help mitigate the burden of firearm injuries. Future research should seek to estimate the direct out-of-pocket and indirect costs of firearm injuries to obtain a more comprehensive understanding of the economic burden.

### Electronic supplementary material

Below is the link to the electronic supplementary material.

Supplementary file1 (DOCX 17 kb)

## Figures and Tables

**Fig. 1:**
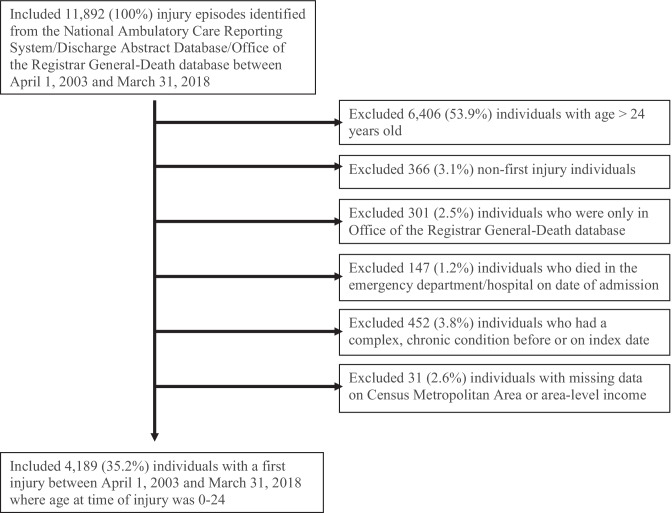
Flowchart of study inclusion and exclusion criteria

## Data Availability

The data from this study are held securely in coded form at ICES. While data sharing agreements prohibit ICES from making the data publicly available, access may be granted to those who meet pre-specified criteria for confidential access, available at www.ices.on.ca/DAS. The full dataset creation plan and underlying analytic code are available from the authors upon request, understanding that the programs may rely upon coding templates or macros that are unique to ICES.

## Abbreviations

CI: Confidence interval
ED: Emergency department
