# Supplementary material for: Healthcare utilization and costs following non-fatal powdered and non-powdered firearm injuries for children and youth
Source: Eur J Pediatr. 2022 Mar 5;181(6):2329–42. doi: 10.1007/s00431-022-04429-4 (PMC9110444; doi:10.1007/s00431-022-04429-4)
Supplement: Supplementary file 1 — Supplementary file1 (DOCX 17 kb) [file 431_2022_4429_MOESM1_ESM.docx]

**Appendix**

**Table A1**. Administrative health care databases in Ontario, Canada

| **Database** | **Setting** | **Description** |
| --- | --- | --- |
| Discharge Abstract Database | acute care hospitalizations | The Discharge Abstract Database is a national database, which contains demographic and clinical data on all acute care inpatient hospitalizations. It also includes psychiatric inpatient hospitalizations for children and adolescents and psychiatric inpatient hospitalizations, which occur in non-psychiatric designated beds. |
| Ontario Mental Health Reporting System | psychiatric hospitalizations | The Ontario Mental Health Reporting System collects demographic and clinical data on all adult psychiatric inpatient hospitalizations in Ontario. |
| Continuing Care Reporting System | complex continuing care, long-term care | The Continuing Care Reporting System contains demographic and clinical information on individuals receiving facility-based continuing care. These services include medical long-term care, rehabilitation, geriatric assessment, respite palliative care, and nursing home care. |
| National Rehabilitation Reporting System | rehabilitation | The National Rehabilitation Reporting System contains national data on rehabilitation facilities and clients, collected from participating adult inpatient rehabilitation facilities and programs. |
| National Ambulatory Care Reporting System | emergency department visits, same-day surgery and outpatient clinic visits | The National Ambulatory Care Reporting System contains data on all ambulatory care including emergency department visits, day surgery and outpatient clinic visits (for example, chemotherapy and dialysis). |
| Ontario Health Insurance Plan Claims Database | physician and outpatient services | The Ontario Health Insurance Plan Claims Database covers all services and procedures provided by health care providers who can claim under the Ontario Health Insurance Plan (such as, physician and laboratory/diagnostic services). |
| Ontario Drug Benefit Claims Database | outpatient prescription drugs | The Ontario Drug Benefit Claims Database includes data on all drugs dispensed in community pharmacies and long-term care/nursing facilities. The Ontario Drug Benefit program covers prescription drugs listed in the provincial formulary for all seniors (aged 65+) as well as those under the age of 65 on social assistance. |
| Home Care Database | home care | The Home Care Database provides data on government-funded services coordinated by Ontario’s Community Care Access Centres for individuals requiring home care. |

**Table A2**. ICD-10 codes used to identify firearm injuries

W32: Handgun discharge (firearm only)

W33: Rifle, shotgun and larger firearm discharge (firearm only)

W3400: Discharge from BB gun

W3401: Discharge from air gun

W3408: Discharge from other specified firearms

W3409: Discharge from unspecified firearm

X93: Assault by handgun discharge (firearm only)

X94: Assault by rifle, shotgun & larger firearm discharge (firearm only)

X9500: Assault by BB gun discharge

X9501: Assault by air gun discharge

X9508: Assault by other specified firearm discharge5

X9509: Assault by unspecified firearm discharge

X72: Intentional self-harm by handgun discharge (firearm only)

X73: Intentional self-harm by rifle, shotgun & larger firearm discharge (firearm only)

X7400: Intentional self-harm BB gun discharge

X7401: Intentional self-harm air gun discharge

X7408: Intentional self-harm other specified firearm discharge

X7409: Intentional self-harm by unspecified firearm discharge

Y22: Handgun discharge undetermined intent (firearm only)

Y23: Rifle shotgun & larger firearm discharge undetermined intent (firearm only)

Y2400: BB gun discharge, undetermined intent

Y2401: Air gun discharge, undetermined intent

Y2408: Other specified firearm discharge, undetermined intent

Y2409: Unspecified firearm discharge, undetermined intent

Y35.0 - Legal intervention involving firearm discharge

**Table A3.** ICD-10 codes to identify firearm injuries by weapon type and intent

**Weapon type**

Handgun

W32: Handgun discharge (firearm only)

X93: Assault by handgun discharge (firearm only)

X72: Intentional self-harm by handgun discharge (firearm only)

Y22: Handgun discharge undetermined intent (firearm only)

Rifle

W33: Rifle, shotgun and larger firearm discharge (firearm only)

X94: Assault by rifle, shotgun & larger firearm discharge (firearm only)

X73: Intentional self-harm by rifle, shotgun & larger firearm discharge (firearm only)

Y23: Rifle shotgun & larger firearm discharge undetermined intent (firearm only)

Non-powdered firearm

W3400: Discharge from BB gun

W3401: Discharge from air gun

X9500: Assault by BB gun discharge

X9501: Assault by air gun discharge

X7400: Intentional self-harm BB gun discharge

X7401: Intentional self-harm air gun discharge

Y2400: BB gun discharge, undetermined intent

Y2401: Air gun discharge, undetermined intent

Other and Unspecified

W3408: Discharge from other specified firearms

W3409: Discharge from unspecified firearm

X9508: Assault by other specified firearm discharge

X9509: Assault by unspecified firearm discharge

X7408: Intentional self-harm other specified firearm discharge

X7409: Intentional self-harm by unspecified firearm discharge

Y2408: Other specified firearm discharge, undetermined intent

Y2409: Unspecified firearm discharge, undetermined intent

Multiple weapons, the following rules apply:

BB*other = BB

Handgun*BB = First use hospitalization record, then use handgun

Handgun*other = Handgun

Handgun*rifle = First use hospitalization and then use handgun

Rifle*BB and Rifle*other = Rifle.

**Intent**

Unintentional firearm injury (Note: in the Office of the Registrar General-Death file only use the first 3 digits W32, W33, W34)

W32: Handgun discharge (firearm only)

W33: Rifle, shotgun and larger firearm discharge (firearm only)

W3400: Discharge from BB gun

W3401: Discharge from air gun

W3408: Discharge from other specified firearms

W3409: Discharge from unspecified firearm

Intentional assault from firearm (Note: in the Office of the Registrar General-Death file only use the first 3 digits X93, X94, X95)

X93: Assault by handgun discharge (firearm only)

X94: Assault by rifle, shotgun & larger firearm discharge (firearm only)

X9500: Assault by BB gun discharge

X9501: Assault by air gun discharge

X9508: Assault by other specified firearm discharge

X9509: Assault by unspecified firearm discharge

Intentional self-harm (self-injury) from firearm (Note: in the Office of the Registrar General-Death file only use the first 3 digits X72, X73, X74)

X72: Intentional self-harm by handgun discharge (firearm only)

X73: Intentional self-harm by rifle, shotgun & larger firearm discharge (firearm only)

X7400: Intentional self-harm BB gun discharge

X7401: Intentional self-harm air gun discharge

X7408: Intentional self-harm other specified firearm discharge

X7409: Intentional self-harm by unspecified firearm discharge

Legal Interventions

Y35.0 - Legal intervention involving firearm discharge

Undetermined (unknown) intent (Note: in the Office of the Registrar General-Death file only use the first 3 digits Y22, Y23, Y24)

Y22: Handgun discharge undetermined intent (firearm only)

Y23: Rifle shotgun & larger firearm discharge undetermined intent (firearm only)

Y2400: BB gun discharge, undetermined intent

Y2401: Air gun discharge, undetermined intent

Y2408: Other specified firearm discharge, undetermined intent

Y2409: Unspecified firearm discharge, undetermined intent

If have multiple intent in one injury episode then using the following rules to decide the intent

Assault*intent unknown = assault

Assault*legal intervention = assault

Legal intervention – keep as separate, do not include in any intents – we just need to enumerate this.

Self-harm*intent unknown = self-harm

Unintentional*assault – assault

Unintentional*assault*legal intervention – assault

Unintentional*intent unknown = unintentional

Unintentional*legal intervention = unintentional

Unintentional*self-harm – self-harm
